# The Evolution of the Safety of Plasma Products from Pathogen Transmission—A Continuing Narrative

**DOI:** 10.3390/pathogens12020318

**Published:** 2023-02-15

**Authors:** Albert Farrugia

**Affiliations:** UWA Medical School, Surgery The University of Western Australia, 35 Stirling Highway, Perth 6009, Australia; albert.farrugia@uwa.edu.au

**Keywords:** plasma products, safety, virology, haemophilia

## Abstract

Chronic recipients of plasma products are at risk of infection from blood-borne pathogens as a result of their inevitable exposure to agents which will contaminate a plasma manufacturing pool made up of thousands of individual donations. The generation of such a pool is an essential part of the large-scale manufacture of these products and is required for good manufacturing practice (GMP). Early observations of the transmission of hepatitis by pooled plasma and serum led to the incorporation of heat treatment of the albumin solution produced by industrial Cohn fractionation of plasma. This led to an absence of pathogen transmission by albumin over decades, during which hepatitis continued to be transmitted by other early plasma fractions, as well as through mainstream blood transfusions. This risk was decreased greatly over the 1960s as an understanding of the epidemiology and viral aetiology of transfusion-transmitted hepatitis led to the exclusion of high-risk groups from the donor population and the development of a blood screening test for hepatitis B. Despite these measures, the first plasma concentrates to treat haemophilia transmitted hepatitis B and other, poorly understood, forms of parenterally transmitted hepatitis. These risks were considered to be acceptable given the life-saving nature of the haemophilia treatment products. The emergence of the human immunodeficiency virus (HIV) as a transfusion-transmitted infection in the early 1980s shifted the focus of attention to this virus, which proved to be vulnerable to a number of inactivation methods introduced during manufacture. Further developments in the field obviated the risk of hepatitis C virus (HCV) which had also infected chronic recipients of plasma products, including haemophilia patients and immunodeficient patients receiving immunoglobulin. The convergence of appropriate donor selection driven by knowledge of viral epidemiology, the development of blood screening now based on molecular diagnostics, and the incorporation of viral inactivation techniques in the manufacturing process are now recognised as constituting a “safety tripod” of measures contributing to safety from pathogen transmission. Of these three components, viral inactivation during manufacture is the major contributor and has proven to be the bulwark securing the safety of plasma derivatives over the past thirty years. Concurrently, the safety of banked blood and components continues to depend on donor selection and screening, in the absence of universally adopted pathogen reduction technology. This has resulted in an inversion in the relative safety of the products of blood banking compared to plasma products. Overall, the experience gained in the past decades has resulted in an absence of pathogen transmission from the current generation of plasma derivatives, but maintaining vigilance, and the surveillance of the emergence of infectious agents, is vital to ensure the continued efficacy of the measures in place and the development of further interventions aimed at obviating safety threats.

## 1. Background—The Development of Plasma-Derived Pharmaceuticals

The derivation of therapeutic products from plasma originates with the discovery and use of blood transfusion. Appreciation of the different properties of the cellular and plasma components of donated blood led to the use of liquid plasma for the treatment of haemorrhage and shock [1]. Concurrently, early developments in immunology led to the use of sera raised in animals against the microorganisms responsible for diphtheria and tetanus in the treatment and pathogenesis of the diseases [2]. Blood and plasma transfusion was also used as an early treatment of haemophilia [3,4]. All these early treatments, covering most of the spectrum of current plasma protein therapeutics, were derived from single or small pools of individual donors of blood or plasma, generally in a hospital setting and very distant from a pharmaceutical manufacturing environment.

**Figure 1:**
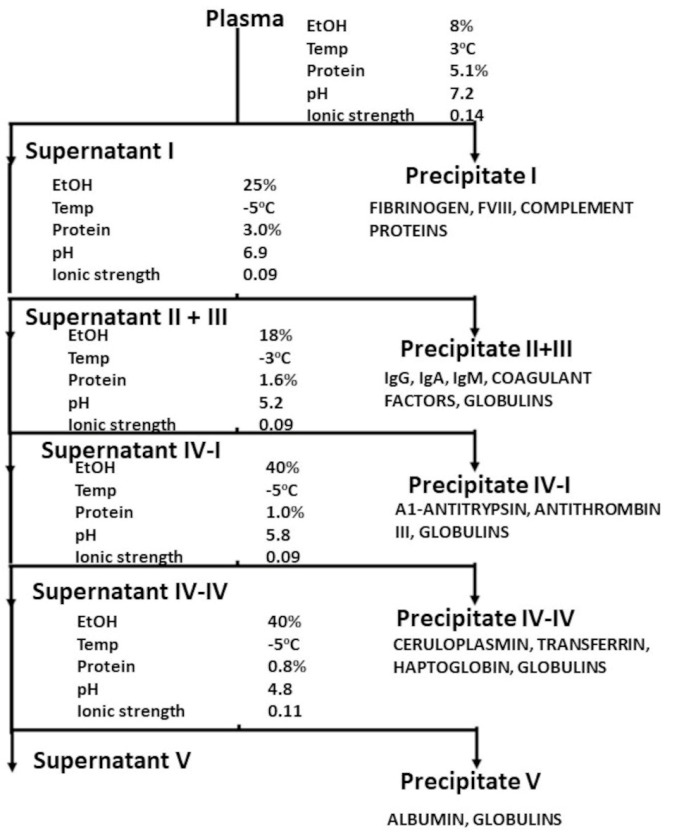
Plasma fractionation using Cohn et al.’s ethanol precipitation technology. From Cohn et al. (1946) with permission.

The industrial production of plasma products has its inception in the fractionation scheme developed by Cohn and his co-workers as the result of the United States military’s interest in a stable plasma substitute to treat blood loss [5,6]. Cohn designed his method so that sequential harvesting of a number of protein fractions could be used to yield a number of potential therapeutic products (Figure 1), in addition to the final albumin product which achieved the project’s aim of an easily administrable plasma substitute [7,8]. These included fibrinogen from Fraction I and immunoglobulin from Fraction II. Albumin’s efficacy in treating battlefield injury was demonstrated with the treatment of burn victims of Pearl Harbour [9], and early studies with fibrinogen concentrates [10] and immunoglobulin [11] demonstrated similar efficacy in conditions characterised by a deficiency in these plasma proteins. All these products of large-scale fractionation were manufactured from large volumes of starting plasma, and composed of pools of many thousands of individual donations. The convergence of a large volume of homogenous raw material with an industrial process resulted in the ability to manufacture large batches of the respective therapeutics, allowing the sampling and characterisation of the products. This resulted in products which could be labelled with the content of the active ingredient in the form of the therapeutic protein, allowing labelling of the administered product and, with further understanding of the pharmacology of these products, accurate dosage and predictable therapeutic effects. These factors allowed the nascent field of plasma protein therapeutics to be established as a significant component of pharmaceutical manufacture. The further development of this sector into its current form is beyond the immediate scope of this paper; the reader is referred to excellent reviews [12,13].

## 2. The (Invisible) Elephant in the (Treatment) Room—The Potential Infection by Pathogens Present in the Plasma Pools

It is intuitively obvious that the probability of including donations from donors harbouring a blood-borne infection increases with the number of donors included in a pool. This scenario has been modelled elegantly by Lynch et al. from the United States Food and Drug Administration (FDA) [14]. Their model derived the relationship between the scale of manufacture (as reflected by the number of donors in a pool), the prevalence of an infectious agent in the donor population and the number of independent treatment episodes. These parameters were used to derive the probability of exposure to an infectious agent. The results are shown in Table 1. Clearly, the probability of exposure for patients undergoing repeated treatments over the course of a period during which an infectious agent is present in the donor population is very high, at the manufactured scale established over the first twenty years of the fractionation industry and maintained ever since.

## 3. Emergence of Pathogen Transmission as a Risk for Recipients of Blood and Plasma Products—The Hepatitis Story

By the 1930s, the only infectious diseases known to be transmitted by blood transfusion were malaria [15] and syphilis [16]. In these early reports, the infections in the donor were frequently undetected. This will be discussed below in relation to viral transmission; in the interim, it should be stated that at no stage have cellular organisms, such as those causing syphilis, malaria, Chagas disease and others, been transmitted by plasma products, due to the elimination of the organisms by the manufacturing process. The extent to which this occurs for other pathogens will be discussed below.

Early observations on the development of jaundice in the recipients of a vaccine containing pooled human lymph [17] were followed by a similar series of outbreaks of jaundice following the administration of vaccines in a pooled plasma or serum matrix (see Table 3 in [18]). Investigation of the largest of these incidents in 1941–1942 revealed that 23 of 970 donors contributing to the serum matrix had a history of jaundice [18]. Concurrently, Beeson [19] reported the transmission of hepatitis by blood and pooled plasma. This growing awareness that pooled, unmodified plasma carried a risk of hepatitis transmission was reviewed in 1947 by Scheinberg et al. [20], who arrived at the conclusions which were the subject of Lynch’s analysis [14] nearly fifty years later, and recommended ceasing the practice of pooling plasma. They also recommended the use of plasma fractions in lieu of unmodified plasma, as these seemed to not transmit hepatitis/jaundice at this time. This latter statement was based on the post-transfusion data available at the time, despite studies showing the retention of viral infectivity in the fractions (unpublished data by Bird et al. cited in [21]. The absence of transmission of hepatitis, or any subsequently emergent blood-borne agent, by albumin fractionated by the Cohn method, has been ascribed to the heat treatment step introduced early on its use [18]. Albumin not subjected to this pasteurisation step transmitted hepatitis [22], showing that the fractionation alone does not eliminate the risk. Subsequent experiments estimated that of 7.5 log_10_ infectious/ml doses introduced in a plasma pool, the fractionation process resulted in a reduction of 5–6 log_10_ infectious doses/ml of hepatitis B, which had been characterised [23] by the time these studies were performed. The residual infectivity was inactivated by pasteurisation [23]. As knowledge of the virology of hepatitis evolved, hepatitis B was identified as a major component of post-transfusion hepatitis, and the screening of blood and plasma donations with successive generations of increasingly sensitive tests for the characteristic surface marker (HBsAg) decreased the incidence of post-transfusion hepatitis [24], but not the transmission of hepatitis B by the products used to treat haemophilia [25,26]; although, most patients overcame the infection and developed protective antibodies to the hepatitis B virus [25]. Concurrently, the FDA’s mandated voluntary blood donation in the USA contributed further to the safety of blood transfusion by excluding high-risk donors from the blood supply [27]; although, this measure was not extended to plasma donors contributing to the pool for plasma products.

By the end of the 1970s, it was recognised that another form of parenterally transmitted hepatitis, designated as “non-N non-B hepatitis (NANBH), was present in the blood and plasma supply. Other screening tests for infectious agents had the effect of decreasing the risk of NANBH [24]. Developments in virology and molecular diagnostics resulted in the characterisation of the virus responsible for most of its transmission by blood, designated as hepatitis C (HCV) [28], and the development of a blood screening test for antibodies associated with the infection. As with HBV, the safety of plasma derivatives followed a different path, and the decrease in the risk of transfusion-transmitted NANBH/HCV was not paralleled by a decrease in the risk to haemophiliacs treated with large-pool concentrates [29,30]. Unlike HBV, HCV infection in the majority of the infected population led to a chronic infection, characterised by cirrhosis and significant morbidity and mortality [31].

## 4. The Catastrophe of AIDS

The effect of the AIDS epidemic on the blood supply has been extensively reviewed [32], as has been the tragic consequence of the epidemic on the recipients of pooled plasma products, mainly the haemophilia community [33]. While the risk from blood transfusion was, as with other viral infections, decreased by excluding high-risk donors through deferral [34], this did not prevent the epidemic in the haemophilia population [35], as a result of the virus contaminating large pools of plasma as subsequently analysed by Lynch [14]. As will be reviewed below, the safety of blood transfusion and plasma products from the risk of HIV transmission converged temporally, for different reasons, so that by the late 1980s, HIV/AIDS faded as a safety threat for plasma product recipients, as may be seen from the CDC’s analysis of birth cohorts in the US haemophilia population [36].

## 5. A Further Note on Pooling

The consequence of pooling plasma donations as a prelude to manufacture has been alluded to frequently in this review. Several reports noted that patient groups which had been treated with products derived from a single donor or small-pool plasma sources had much smaller rates of infection of transmitted transfusion viruses than similar patients treated with large-pool products [37,38,39,40,41]. These findings indicate that the pooling of donations was fundamental to the safety of haemophilia treatment products, even in products sourced from voluntary unpaid donors. This is supported by the experience in the haemophilia community in Australia [30].

## 6. The Development of Effective Measures for Assuring Plasma Product Safety

Epidemiological studies indicate that the prevalence of HIV in the US population in 1982–83—the period over which donor deferral measures excluding AIDS-high-risk groups were implemented—was ca 1.2/1000 population; this rate dropped to ca 1.7/1000 population in 1984, by which year deferral of high-risk groups was well established [34,42]. The analysis of Lynch et al. (Table 1) referred to above indicates that, as this prevalence, with a plasma pool size as utilised then (and now), the exposure of patients, such as haemophiliacs undergoing several treatments, approaches 100%. However, and undoubtedly, helpful these deferral measures were for single donor transfusions [34], they had a minimal effect on the safety of pooled plasma products. The first HIV screening test introduced in 1985 had a sensitivity of 99% [43], suggesting that this would have reduced the prevalence of HIV by two log_10_ of virus, to ca 1.2/100,000 donations. This indicates that for intensively treated patients, such as haemophiliacs on prophylactic regimens which were underway, albeit in a limited fashion, by the early 1980s [44], a continued risk of exposure to HIV would have ensued. This has been confirmed in other analyses [45,46].

This realisation accelerated efforts to develop viral inactivation methods for eliminating the risk of HIV (reviewed by Foster [18,47]). Following decades of demonstrated safety by pasteurised albumin, early efforts focussed on enhancing the safety of Factor VIII concentrate from hepatitis transmission using this method, with the additional feature of adding excipients to protect factor VIII from inactivation [48]. The low Factor VIII yield obtained with this method precluded its wide adoption, but the accelerated development spurred by AIDS led to the adoption of a number of heat treatment processes, mostly on the final dried and vialed product, which employed temperatures of ca. 60 °C. These products were generally effective in inactivating HIV and preventing AIDS in haemophiliacs [49,50] but did not prevent the transmission of NANBH [51]. Raising the temperature for heating the dried Factor VIII to 80 °C was effective in this regard [52,53].

**Figure 2:**
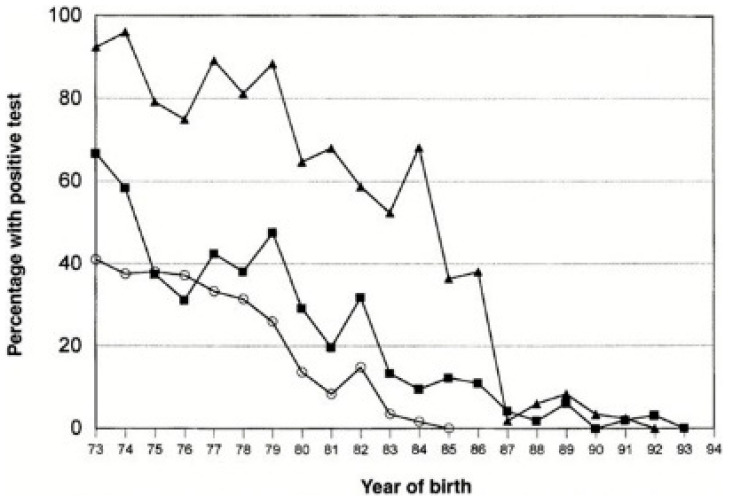
Prevalence of blood-borne infections in haemophilic birth cohorts in the United States. Based on the results of laboratory testing for HBV (▪), HCV(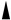) and HIV-1 (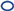). The proportion was zero for HIV after 1984, for HCV after 1992, and for HBV after 1993. From Soucie et al. 2001, used with permission.

In 1985, Horowitz and colleagues at the New York Blood Center published their first results on the inactivation of lipid-enveloped viruses using solvent-detergent mixtures [54], a technology which rapidly rose to the status of a benchmark in ensuring safety from the main blood-borne pathogenic viruses HIV, HCV and HBV [55]. This enhanced greatly the safety of products for the haemophilia population (Figure 2). It is therefore all the more unfortunate that such measures were not immediately implemented for all plasma products. Early preparations of immunoglobulin prepared by Cohn’s main Method 6 resulted in a 16% solution of protein, which had to be administered intramuscularly or subcutaneously because intravenous administration led to severe systemic reactions in some patients; some preparations did not transmit hepatitis [56,57]. Retrospectively, this can be seen to have induced a level of complacency on the safety of immunoglobulins in industry and regulators alike, despite occasional reports of unspecified hepatitis being transmitted by intramuscular immunoglobulin [58,59,60]. The issue was brought back into focus when, with the development of intravenously administered immunoglobulin solutions over the 1970s, NANBH/HCV was transmitted by some of these products. Of particular note are two epidemics—one in East Germany prior to German reunification and one in Ireland—in which rhesus-negative women were given anti-RhD immunoglobulin post-partum which transmitted NANBH/HCV to hundreds of healthy women [61,62]. In both instances, donors with a history of jaundice were included in the plasma pool, which was fractionated into anti-RhD immunoglobulin using chromatography rather than Cohn fractionation. In addition, and unusually for that period, both preparations were administered intravenously. The possible role of manufacturing methods in the transmission of NANBH/HCV has been described [63,64], and, while most of the reported transmissions have been with products manufactured with some deviations from potentially viricidal steps in the Cohn method, the finding that Cohn Fraction II, from which all immunoglobulin products were derived, which is the parent fraction of all immunoglobulin products, contained HCV viral nucleic acid [65,66], indicated that the long-standing comfort in the safety of immunoglobulin derived from Cohn fractionation had been misplaced. Yu et al. [67,68] have postulated that the removal of anti-HCV-complexing antibodies through early generations of anti-HCV screening tests altered the properties of HCV present in the plasma pool and led to free, uncomplexed HCV partitioning into Fraction II, rather than precipitating, as an antibody–virus complex, into Fraction III, resulting in infectious products. Countering this hypothesis, it should be noted that the first reports of HCV infective intravenous immunoglobulin occurred prior to the introduction of screening tests [69]. Irrespective of the mechanism, there can be no doubt that the failure to mandate and implement viral inactivation methods for immunoglobulin products when these became available in the mid-1980s, particularly solvent–detergent treatment which would have eliminated any threat of HCV, represents a significant collective failure on the part of regulators and industry alike, such methods were universally adopted from 1995 on, leading to the current status of immunoglobulin safety.

## 7. A Footnote—Safety from Prion Transmission

The role of prions in the aetiology of transmissible spongiform encephalopathies (TSEs) has been extensively reviewed [70]. Attention in the haematological sector has focussed on Creutzfeldt–Jakob Disease (CJD) and variant Creutzfeldt–Jakob Disease (vCJD), a type of TSE caused by the consumption of beef contaminated by the bovine TSE Bovine Spongiform Encephalopathy (BSE). A hypothetical risk of transmission through blood and blood products could be present if exposed to the putative infectious agent [71,72]. Several epidemiological studies show no evidence of the transmission of classical CJD by blood transfusion [73]. The presence of blood infectivity of vCJD predicted by tissue localisation and large animal models [74] was apparently confirmed by reports of four transmissions through blood transmissions in the early 2000s [75]. No cases have been detected since 2007. Studies on the partitioning of the infectious agent during plasma fractionation indicated that intermediate-purity Factor VIII concentrate productions schemes had a limited ability to partition the infectivity away from the therapeutic fraction [76], while extensive removal of infectivity was observed during modelled fractionation to immunoglobulin and albumin [72]. This is supported by evidence of transmission of vCJD, manifested sub-clinically, in a haemophiliac given intermediate-purity Factor VIII manufactured from a plasma pool including a donation from a donor who subsequently developed vCJD [77]. An immunoglobulin concentrate derived from a similarly contaminated plasma pool did not show evidence of transmission of the infective agent [78]. The blood industry and its regulators have relaxed the measures relating to vCJD, to the extent that plasma collected from donors who were potentially exposed to BSE during the period of the epidemic in the UK are once again allowed to donate plasma in the UK and the USA [79,80]. It remains to be seen whether TSEs will be resurrected as a threat to the safety of blood-derived therapies; the reader is referred to an excellent review [81] which assesses the various dimensions underpinning this area.

## 8. Development of a Cohesive Framework—The Current Landscape of Plasma Product Safety

This review has described how the evolution of the safety of blood and blood components has centred on the deferral of donors at risk of infection based on disease epidemiology and the development of screening tests to exclude any infected donations. It should be clear by now that these measures have had a limited effect on the safety of plasma products, and that the elimination of the infectious agents which contaminate a plasma pool has proven to be pivotal for these products. This is further augmented when considering the plethora of emerging and re-emerging infectious agents, particularly arboviruses, which have threatened the blood supply over the past twenty years [82]. A number of human and ecological factors have contributed to this phenomenon, including global travel, environmental destruction, agricultural practices and others [83,84]. A number of reports have described the transmission of these emergent infections by blood and component transfusion [85]. There have been no reports of such transmissions by plasma derivatives produced over the past thirty-year era of robust pathogen-reduction methods, despite the undoubted presence of many of the agents in the plasma pool, given that source plasma from the USA, the source of two-thirds of the global plasma supply, is not screened for these agents. Kreil’s group has validated repeatedly the inactivation process’ capacity to eliminate these agents [86,87,88]. In addition, while the donor selection and testing measures in place for blood for transfusion have been very effective, rare transmissions of the main transfusion-transmitted viruses are still reported, even after implementation of all these measures [89,90,91]. No such transmissions have occurred through plasma derivatives.

Hence, the concept of the “Tripod” of safety measures proposed conceptually by this author in 2004 [92] has been amended elegantly by Kreil to emphasise that, of the three elements of this “tripod”—donor selection, donation testing and pathogen reduction—it is the systematic inclusion of robust pathogen inactivation/removal steps in the manufacturing process which have brought the safety of plasma derivatives to its current status [93]. In the case of fresh blood components, this fundamental element of the “tripod” is still lacking in most transfusion practices. Although technologies for effective pathogen reduction have been developed for plasma for transfusion and for platelets [94], their adoption has not been mandated by the main regulatory agencies, and their uptake has been limited to specific countries, frequently during topical, restricted outbreaks of infectious agents. It is to be hoped that this apparent lassitude does not influence blood safety when further epidemics arise. This author has heard regulatory officials assert that pathogen reduction for components is of limited value when technology for pathogen-reduced red cells is as yet unavailable, and it remains to be seen if this attitude changes when such a technology is available in the not-distant future. In this context, the recommendation of the United States Institute of Medicine, to the FDA, in its seminal report on the safety of the blood supply [95] should be borne in mind by today’s decision makers:

“Where uncertainties or countervailing public health concerns preclude completely eliminating potential risks, the FDA should encourage, and where necessary require, the blood industry to implement partial solutions that have little risk of causing harm”.

## 9. Concluding Reflections

This review has attempted to follow the path towards the current high level of safety of plasma products. This path has been a long and difficult one, and, despite the necessarily linear nature of the temporal developments described in this review, has not been entirely linear in reality. As proposed by Murphy [96], the complex nexus of factors affecting blood safety render this landscape unpredictable, a feature which is easily perceived when one considers, for example, the factors contributing to the emergence of pathogens due to environmental destruction [83]. Hence, the contributions to safety described in this review, in particular, pathogen reduction during manufacture, have to be maintained in their current robust states and, if anything, further enhanced, in order to continue to keep plasma products safe. This review contends that at specific points in this progression, a collective failure to appreciate the risks of established or emerging pathogens contributed to measures being delayed or ignored. It is to be hoped that the lessons from these failures, still visible through ongoing judicial processes [97], will contribute to ensuring that they will not be repeated.

## Figures and Tables

**Table 1:** Effect of manufacturing scale on risk of exposure. Adapted from Lynch et al. 1996 with permission.

Manufacturing Scale (Number of Donors)	Number of Independent Infusions		
	1	10	100
Prevalence of agent = 5 × 10^−5^			
60,000	11%	70%	100%
25,000	2%	18%	86%
1000	0.2%	2%	18%
Prevalence of agent = 5 × 10^−4^			
60,000	70%	100%	100%
25,000	39%	99%	100%
1000	2%	18%	86%
Prevalence of agent = 5 × 10^−3^			
60,000	100%	100%	100%
25,000	99%	100%	100%
1000	18%	86%	100%
